# Central Sensitization and Its Role in Persistent Pain Among Spondyloarthritis Patients on Biological Treatments

**DOI:** 10.3390/medicina61020319

**Published:** 2025-02-12

**Authors:** Nuran Öz, Aygün Özer, Mehmet Tuncay Duruöz

**Affiliations:** 1 Physical Medicine and Rehabilitation Department, Rheumatology Division, Marmara University School of Medicine, 34899 Istanbul, Türkiye; tuncayduruoz@gmail.com; 2 Physical Medicine and Rehabilitation Department, Rheumatology Division, Abant Izzet Baysal Training and Research Hospital, 14280 Bolu, Türkiye; dr.aygun.aliyeva@gmail.com

**Keywords:** central sensitization, spondyloarthritis, biological treatments, disease activity

## Abstract

*Objectives*: Spondyloarthritis (SpA) is a chronic inflammatory arthritis that mainly affects the sacroiliac joints and spine. Despite effective biological treatments, persistent pain is common in SpA patients, potentially due to central sensitization (CS), a condition of heightened central nervous system responsiveness. The purpose of this study was to investigate the link between disease activity and CS in SpA patients on biological therapy. *Patients and Methods*: One hundred and twenty SpA patients with at least six months of treatment with biological agents were included in this cross-sectional study. Patients’ demographic, clinical, and functional information were collected. The assessment of CS was conducted using the Central Sensitization Inventory (CSI), whereas disease activity and quality of life were evaluated using the Bath Ankylosing Spondylitis Disease Activity Index (BASDAI), Ankylosing Spondylitis Disease Activity Score (ASDAS)-C-reactive protein (CRP), and Ankylosing Spondylitis Quality of Life (ASQoL). Statistical analyses included correlation assessments and logistic regression to identify predictors of CS. *Results*: CS (CSI ≥ 40) was present in 40.8% of patients. Disease activity was significantly higher and quality of life was lower in patients with CS. BASDAI and ASQoL scores were strongly correlated with CS (r = 0.774 and r = 0.839, respectively). Logistic regression identified ASQoL and BASDAI scores as independent predictors of CS. ROC curve analysis demonstrated that ASQoL had the highest discriminative ability for predicting CS (AUC = 0.97). *Conclusions*: CS is significantly associated with higher disease activity and poorer quality of life in SpA patients receiving biological therapy. Incorporating CS assessment into routine clinical practice may enhance our understanding and management of persistent symptoms in SpA, improving patient outcomes.

## 1. Introduction

Spondyloarthritis (SpA) is a chronic autoimmune inflammatory arthritis that can mainly affect the sacroiliac joints but also the whole spine and peripheral joints, which are characterized by inflammatory back pain that improves with movement and worsens with rest, stiffness, and enthesitis [1,2]. Disease activity in SpA, which reflects the severity of symptoms and inflammation, is crucial for treatment planning and monitoring patient outcomes. It is commonly assessed using validated indices such as the Bath Ankylosing Spondylitis Disease Activity Index (BASDAI) and Ankylosing Spondylitis Disease Activity Score (ASDAS), which incorporate both subjective symptoms and objective inflammatory markers [3]. Biomarkers such as HLA-B27, C-reactive protein (CRP), and the erythrocyte sedimentation rate (ESR) are commonly used biomarkers for the diagnosis of axial spondyloarthritis (axSpA) and are corroborated by imaging techniques such as MRI and X-rays. The treatment generally starts with nonsteroidal anti-inflammatory drugs (NSAIDs) to control pain and inflammation, while biologics such as tumor necrosis factor (TNF) and interleukin-17 (IL-17) inhibitors are recommended for patients with persistent disease activity. While these treatments are effective, they can cause side effects such as gastrointestinal and renal problems with NSAIDs and increased risk of infection with biologics [4]. The main goal of treatment is to improve symptoms, especially pain, control inflammation, and prevent progressive structural damage in order to maintain the patient’s daily functioning and social participation [5]. Pain in SpA is mostly attributed to inflammation; intense and disabling pain persists despite treatment with powerful biological agents that control inflammation [6]. Recently, it is considered that central sensitization (CS) is a possible cause of these persistent symptoms [7].

CS has been defined as ‘hyperexcitability of the central nervous system (CNS)’. This is the ongoing increase in the sensitivity of nociceptive neurons within the CNS to sensory signals coming from peripheral sources [8]. In recent studies, CS has been reported in various rheumatological diseases such as fibromyalgia, osteoarthritis, rheumatoid arthritis, ankylosing spondylitis, and systemic lupus erythematosus [9]. As a consequence of neuroinflammation in the peripheral and CNS, a burst of proinflammatory cytokines and chemokines released from activated microglia and astrocytes in the brain and spinal cord leads to widespread changes in pain processing pathways. This neuroimmune response amplifies nociceptive signals, enhances synaptic plasticity, and disrupts the normal balance of excitatory and inhibitory mechanisms in the CNS. Additionally, the downregulation of inhibitory neurotransmitters like gamma-aminobutyric acid (GABA) and glycine, coupled with increased activity of excitatory mediators such as glutamate and substance P, perpetuates the hypersensitivity. These changes contribute to generalized pain, hyperalgesia, and allodynia, which are hallmark features of CS [10,11,12].

The purpose of this study was to evaluate the relationship between disease activity and CS in patients with SpA undergoing biological therapy. Despite the fact that biological therapies effectively control inflammation and reduce disease activity, many patients continue to experience chronic pain, suggesting the involvement of CS as an important mechanism in pain [13]. By addressing both the inflammatory and non-inflammatory components of pain, understanding this relationship may help to optimize treatment strategies as well as improve personalized care and overall patient outcomes.

## 2. Materials and Methods

### 2.1. Patients and Study Design

One hundred and twenty SpA patients (71 males and 49 females) who met the Assessment of the SpondyloArthritis International Society (ASAS) 2009 criteria and who had been treated with biological agents for at least 6 months were included in this cross-sectional observational study [14]. The patients were informed of the study’s purpose and evaluation methods, and informed consent was obtained from those who agreed to take part. The exclusion criteria were: patients with other systemic inflammatory rheumatic diseases, peripheral vascular diseases, peripheral neuropathies, malignancy, neurological or psychiatric diseases and spinal diseases (e.g., spinal stenosis), patients on centrally acting pain relievers including duloxetine, opioid, and pregabalin, and those with a previous diagnosis of fibromyalgia (FM) syndrome and/or neuropathic pain. Approval for the study was granted by the Marmara University Faculty of Medicine Research Ethics Committee, approval number 09.2024.973, and the research was conducted in compliance with the Declaration of Helsinki.

### 2.2. Assessment of Central Sensitization and Pain

CS was assessed using the Central Sensitization Inventory (CSI), a self-reported questionnaire designed to identify patients with symptoms initially associated with CS. The first part of this two-part scale consists of 25 items that examine emotional and somatic disturbances associated with CS. The CSI assesses 25 questions covering seven main domains: pain and somatic symptoms, autonomic nervous system dysregulation, fatigue and sleep disturbances, cognitive and emotional dysregulation, sensory sensitivity, gastrointestinal and visceral symptoms, and the emotional and psychological impact of chronic conditions. Thus, the CSI provides a comprehensive assessment of the multidimensional impact of CS including physical, emotional, and cognitive aspects that not only focus on pain, but also collectively reflect the complexity of the syndrome. Items are evaluated on a 0 to 4 scale, with the overall score varying from 0 to 100. A CSI score of 40 or higher in individuals with chronic pain suggests a strong possibility of CS [6]. The second section of the questionnaire inquires whether there has been any prior diagnosis of a condition linked to CS syndrome [15]. High scores are associated with severe symptomatology. We used this inventory, which has been validated in Turkish [16].

Using the Visual Analog Scale (VAS), pain intensity was rated by patients on a scale from 0 (no pain) to 10 (the most severe pain conceivable) [7].

### 2.3. Data Collection

Information was gathered on sociodemographic data, body mass index (BMI), smoking and alcohol consumption, time since diagnosis (in years), symptom duration (in years), duration of morning stiffness (in minutes), medical history, comorbidities, history of extra-articular manifestations, and treatment history through patient interviews and medical records. ESR (mm/h), CRP (mg/L), and vitamin D levels (ng/mL) were noted from routine controls. Whole blood samples for ESR were collected into EDTA tubes and analyzed using the Westergren method, which measures the sedimentation rate of red blood cells over one hour. CRP levels were quantified from the serum samples using a high-sensitivity immunoturbidimetric assay on an automated analyzer. Levels of 25-hydroxyvitamin D were measured in serum samples using a chemiluminescence immunoassay (CLIA). A total of 120 blood samples were analyzed, collected from patients who met the inclusion criteria of the study. In the present study, a cross-sectional design was used to compare SpA patients with CS (CSI ≥ 40) and patients without central susceptibility (CSI < 40). Classification of disease activity, ESR, CRP, and other parameters as lower or higher was based on group comparisons within the study population.

AxSpA refers specifically to a subset of SpA characterized predominantly by inflammation in the axial skeleton including the sacroiliac joints and spine. We assessed disease activity using the Bath Ankylosing Spondylitis Disease Activity Index (BASDAI) and ASDAS-CRP [17,18]. The Bath Ankylosing Spondylitis Functional Index (BASFI) and Bath Ankylosing Spondylitis Metrology Index (BASMI) were also used for disease assessment [19,20]. The Maastricht Ankylosing Spondylitis Enthesitis Score (MASES), in which thirteen enthesal sites were evaluated, was used to assess the enthesal involvement of the patients, and the total score ranged from a minimum of 0 to a maximum of 13 [21]. The general health status of the patients in the last week, pain, and fatigue were evaluated using the Visual Analog Scale (VAS) between 0 and 10. Physician’s Global Assessment was also performed with this method. The Ankylosing Spondylitis Quality of Life Questionnaire (ASQoL) was utilized, which consists of 18 questions with a score of ‘1’ or ‘0’ for each question. The total score ranged from 0 to 18, with higher scores indicating poorer quality of life [22].

### 2.4. Statistical Analysis

Data were analyzed using Statistical Package for the Social Sciences (SPSS) version 26.0 (IBM, Chicago, IL, USA). The Shapiro–Wilk test was used to evaluate the normality of all continuous variables and it was accepted that the distribution was normal if a *p* > 0.05 value was obtained, and the distribution was not normal if a *p* < 0.05 value was obtained. It was found that the distribution of age, weight, CSI Part A, ASDAS-CRP, and ASDAS-ESR was normal, and the distribution of all other continuous variables was not normal. Among the continuous variables, those with normal distribution were expressed as the mean ± standard deviation (SD) and those without a normal distribution were expressed as the median [25% (Q1)–75% (Q3) quartile], while categorical variables were reported as the number and percentage. For the comparison of continuous variables, the independent-samples *t*-test was used for those with normal distribution, and the Mann–Whitney U test was applied for those without normal distribution. Differences in categorical variables were evaluated using the chi-square test or Fisher’s exact tests for categorical variables with frequencies less than 5. In the correlation analysis between the CSI score and clinical variables, the Pearson correlation method was used for normally distributed data, while the Spearman correlation method was used for non-normally distributed data. Correlation values were reported as rho (ρ), with rho > 0.80 indicating a very high correlation, 0.60 to 0.80 indicating a high correlation, 0.40 to 0.60 indicating a moderate correlation, 0.20 to 0.40 indicating a weak correlation, and < 0.20 indicating a very weak correlation. All continuous and categorical variables were included in univariate binary logistic regression (LR) analysis to determine independent associations for CSI ≥ 40, respectively. Then, all variables with a *p* value < 0.05 in the univariate analysis were included in the multivariate LR analysis, and predictive variables for CSI ≥ 40 were determined. Odds ratios (ORs) and 95% confidence intervals (CIs) were calculated. Receiver operating characteristic (ROC) curve analyses were conducted to determine the clinical variables most predictive of a CSI ≥ 40, based on specificity and sensitivity. A *p*-value of < 0.05 was considered statistically significant.

## 3. Results

### 3.1. Patients’ Characteristics

In total, 120 axSpA cases on biologic drugs were included in this study. The mean age of the patients was 43.9 ± 9.24 years and 40.8% (n = 49) was female. The median BMI (kg/m^2^) was 26.6 (24–29.1), and 26.7% of the patients was obese. The radiographic axSpA patient rate was 72.5% (n = 87). Regarding the use of biological drugs, the three most frequently used drugs were adalimumab, golimumab, and sekukinumab at 28.3%, 26.7%, and 19.2%, respectively. Patients using ≥3 biological drugs were 10.8% (n = 13). The mean CSI was 35.44 ± 16.25, the median BASDAI score was 3.4 (2.5–5.4), and the median ASQoL score was 6 (4–11). All demographic and clinical parameters are given in Table 1 and Table 2. The study population was divided into two groups as CSI < 40 and CSI ≥ 40 [6,7,8].

### 3.2. Comparisons Between Patients CSI < 40 and CSI ≥ 40

Socio-demographic characteristics, clinical features, comorbidity, disease-related clinical variables, and laboratory findings of patients with CSI < 40 and CSI ≥ 40 scores were compared and are given in Table 1 and Table 2. No significant difference was found between the groups in terms of age, gender, BMI, marital status, education, current smoker, type of axSpA (radiographic or non-radiographic), comorbidity (hypertension, diabetes mellitus and obesity), hip involvement, history of uveitis, inflammatory bowel disease (IBD) and psoriasis, disease duration, biologic drugs used, BASMI score, CRP, ESR, and 25-hydroxy-vitamin D. On the other hand, the duration of morning stiffness and patients on ≥3 biologic medications were significantly higher, while symptom duration and diagnostic latency were lower in patients with CSI ≥ 40. In this study, diagnostic latency refers to the time interval between the onset of symptoms and the formal diagnosis of spondyloarthritis (SpA). A shorter diagnostic latency was observed in patients with CSI ≥ 40. Furthermore, disease-related variables [BASDAI, ASDAS-CRP, ASDAS-ESR, BASFI, MASES, ASQoL, VAS-fatigue, VAS-pain, Patient Global Assessment (PtGA) and Physician Global Assessment (PGA) score] patients with CSI ≥ 40 were significantly worse (*p* < 0.05 for all) (Table 2).

### 3.3. Results of Correlation Analysis

The correlation of disease-related clinical variables with CSI score is given in Table 3. BASDAI score (r = 0.774; *p* < 0.01) and VAS-fatigue score (r = 0.666; *p* < 0.01) were highly correlated with CSI. A very high (r = 0.839; *p* < 0.01) significant positive correlation was found between ASQoL score and CSI.

### 3.4. Results of Logistic Regression Analysis (Predictors of CSI ≥ 40)

All categorical and continuous variables were included in the univariate binary logistic regression analysis to determine whether there was an effect on CSI ≥ 40, respectively. The significant results for the univariate model were as follows, respectively: patients on ≥3 biological medications (OR 22.703, 95% CI 2.840–181.456), BASDAI score (OR 3.943, 95% CI 2.540–6.210), ASDAS-CRP (OR 4. 323, 95% CI 2.241–8.340), ASDAS-ESR (OR 3.529, 95% CI 1.948–6.394), BASFI score (OR 1.792, 95% CI 1.428–2.247), MASES score (OR 1. 164, 95% CI 1.029–1.316), ASQoL score (OR 3.165, 95% CI 2.034–4.924), VAS-fatigue (OR 1.072, 95% CI 1.046–1. 100), VAS pain (OR 1.077, 95% CI 1.050–1.106), PtGA score (OR 1.028, 95% CI 1.009–1.048) and PGA score (OR 1.023, 95% CI 1.004–1.043). Then, in the multivariate model with the inclusion of all variables that were significant in the univariate analysis, in the analysis using the backward LR method: only ASQoL score (OR 3.030, 95% CI 1.755–5.230) and BASDAI score (OR 2.221, 95% CI 1.134–4.350) were statistically significant and these variables are shown in Table 4.

### 3.5. The Results of ROC Analysis

**Figure 1:**
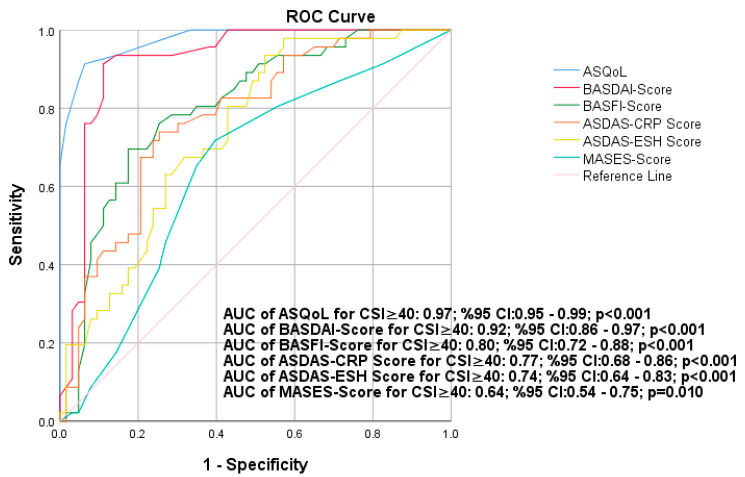
In the ROC analysis, the area under the ROC curve value of ASQoL, BASDAI score, BASFI score, ASDAS-CRP score, ASDAS-ESR score, and MASES score for CSI ≥ 40 indicates the ROC, receiver operating characteristic; ASQoL, Ankylosing Spondylitis Quality of Life Questionnaire; BASDAI, the Bath Ankylosing Spondylitis Disease Activity Index; BASFI, Bath Ankylosing Spondylitis Functional Index; ASDAS, Ankylosing Spondylitis Disease Activity Score; CRP, C-reactive protein; ESR, erythrocyte sedimentation rate; MASES, Maastricht Ankylosing Spondylitis Enthesitis Score; CSI, Central Sensitization Inventory.

In ROC analysis, area under the ROC curve value of ASQoL, BASDAI score, BASFI score, ASDAS-CRP score, ASDAS-ESR score, and MASES score for CSI ≥ 40 were 0.97 (0.95–0.99 95% CI, *p* < 0.001), 0. 92 (0.86–0.97 95% CI, *p* < 0.001), 0.80 (0.72–0.88 95% CI, *p* < 0.001), 0.77 (0.68–0.86 95% CI, *p* < 0.001), 0.74 (0.64–0.83 95% CI, *p* < 0.001), and 0.64 (0.54–0.75 95% CI, *p* < 0.001), respectively (Figure 1). The best cut-off values for ASQoL, BASDAI score, BASFI score, ASDAS-CRP score, ASDAS-ESR score, and MASES score obtained by the ROC curve analysis were ≥6.5 (sensitivity: 93.5%, specificity: 85.7%), ≥3.75 (sensitivity: 93.5%, specificity: 85.7%), ≥3.45 (sensitivity: 80.4%, specificity: 65.1%), ≥2.73 (sensitivity: 76.1%, specificity: 69.8%), ≥2.73 (sensitivity: 80.4%, specificity: 57.1%), and ≥4.5 (sensitivity: 65.2%, specificity: 65.1%) for the prediction.

## 4. Discussion

This study demonstrates that CS is prevalent among patients with SpA receiving biological therapy, with 40.8% of patients exhibiting a CSI score ≥ 40. We also showed that CS was significantly associated with higher disease activity in patients with SpA receiving biological therapy. Patients with CS had significantly higher disease activity scores (BASDAI, ASDAS-CRP), poorer quality of life (ASQoL), and more severe symptoms (VAS-fatigue and VAS-pain) compared with those without CS. Logistic regression analysis identified ASQoL and BASDAI scores as independent predictors of CS, highlighting their role in assessing symptom burden. The ROC curve analysis showed that ASQoL had the highest discriminative ability for CS (AUC = 0.97), emphasizing the importance of quality-of-life assessments in routine care.

CS, which is characterized by hyperexcitability of the CNS, amplifies nociceptive signaling and contributes to chronic pain. This mechanism involves neuroinflammatory changes including the release of cytokines and chemokines, which influence both the peripheral and central pathways [11,12]. The observed correlation between CSI and disease activity scores, such as BASDAI and ASDAS-CRP, indicates that persistent pain in axSpA may arise not only from inflammation, but also from non-inflammatory factors like CS. These findings underscore the importance of comprehensive assessments that account for both inflammatory and central pain mechanisms to better understand and manage the complexities of SpA symptoms.

The correlation of disease-related clinical variables with the CSI score was very highly correlated with the ASQoL score and highly correlated with the BASDAI score, VAS-fatigue score, and VAS-pain score. Based on multivariate analysis, high ASQoL score and BASDAI score were found to be predictive among some possible factors associated with the presence of CS. Notably, this suggests that CS may play a role in the persistence of symptoms in these patients, even when they receive a treatment strongly aimed at reducing inflammation. Incorporating CS assessment into routine clinical practice may help to better understand and manage the complex pain mechanisms in SpA, and ultimately improve patient outcomes.

Pain in patients with axSpA is usually caused by axial inflammation or structural changes based on new bone formation. However, even with powerful biological agents that control inflammation, pain is not always controlled. Pain is multifactorial, with CS being one of the most important factors [23]. CS, defined as amplification of neural signaling pathways, results in hypersensitivity to pain in the CNS with chronic pain signaling in many rheumatic diseases [24]. Despite effective anti-inflammatory treatment, persistent symptoms in SpA may be driven by CS. The study’s findings, particularly the strong correlation between CSI and ASQoL, suggest that CS contributes to pain and reduced quality of life. This emphasizes the importance of identifying patients with high CSI scores and incorporating non-inflammatory pain management strategies such as pharmacological treatments targeting central pain mechanisms and non-pharmacological interventions like cognitive-behavioral therapy. In a study evaluating CS in axSpA, 45% of patients had a CSI score of 40 and above [8]. The prevalence of CS was found to be 46.6% in axSpA patients and 13.7% in the controls in another study comparing CS and FM in axSpA cases with healthy controls [25]. In our study evaluating patients with spondyloarthropathy under biological treatment, CS was found in 49 out of 120 patients with a rate of 40.8%, which is consistent with the literature [8,25]. In a study in which cases with AxSPA were evaluated regardless of the treatment agent (NSAIDs or biological agents), the presence of CS according to CSI was present in 60 out of 100 axSpA patients (60% of patients). This difference with our study may suggest the possible CS effect of biological agents or that ongoing inflammation increases CS [5].

Kieskamp et al., in their study on CS in 100 axSpA patients, found CS in approximately 50% of patients and also found that the presence of CS was associated with a higher quality of life score measured by ASQoL. This indicates greater physical and emotional burden compared with those without CS. This finding emphasizes the impact of CS on overall patient well-being [8]. In our study, quality of life assessed by ASQoL was found to be significantly different between the two groups, with higher scores and worse quality of life in patients with CS. Moreover, while treatment with powerful biological agents that manage disease inflammation and pain in SpA is effective, it does not always alleviate fatigue symptoms in these patients. In 129 patients evaluated after 10 weeks of anti-TNF treatment, 60% of patients showed improvement in pain, although a smaller proportion of patients, 22%, showed improvement in both pain and fatigue [26]. We found significantly higher VAS-pain and VAS-fatigue values in the high CSI group compared with the other group in our study, suggesting that CS has an important effect on the ongoing pain and fatigue in patients under all biological treatments. In support of our results, fatigue and quality of life were found to be significantly worse in the group with CS in the study by Yücel et al. [5]. In a study in which fatigue, pain, and central sensitivity were also evaluated in patients with musculoskeletal pain, fatigue was found to be associated with central sensitivity independently of the presence of pain [27]. Furthermore, persistent symptoms can also be exacerbated by factors such as irregular eating patterns, unhealthy diets, medication non-compliance, and environmental triggers. These lifestyle factors can influence systemic inflammation and pain perception and potentially aggravate CS. Addressing these factors through patient education on maintaining healthy lifestyles, adhering to prescribed treatments, and managing environmental stressors can improve overall disease management and reduce the effects of CS. Combining lifestyle changes with medical treatment can help achieve better outcomes for patients with SpA.

By identifying ASQoL and BASDAI scores as independent predictors of a CSI ≥ 40, our findings underscore the significant role these factors play in the persistence of symptoms, despite effective anti-inflammatory treatment. The results of the present study demonstrated that ASQoL and BASDAI were independent risk factors for CS in the multivariate model. In one of the important results of our study, CS was detected in 12 out of 13 patients with a history of use of 3 or more biological agents according to the CSI. Patients who are evaluated as multiple biological non-responders should be evaluated for the presence of CS, and CS treatment should be planned in addition to their current treatment in patients with CS. These results were consistent with the studies by Şaş et al. and Kieskamp et al., who found that ASQoL was associated with CS in axSpA [8,25]. This suggests that higher disease activity and poorer quality of life are closely linked to the likelihood of CS, which could, in turn, affect the treatment outcomes. The ROC curve analysis further corroborates these findings by demonstrating the predictive power of these variables, with the ASQoL score showing the highest area under the curve (AUC) value of 0.97. This high AUC indicates excellent discriminative ability, confirming the robustness of ASQoL as a predictor for CS in this patient population. The identification of optimal cut-off values for these variables also provides practical clinical thresholds that can guide more personalized patient management, particularly in those who may be at risk of being misclassified as non-responders to biological therapy due to the influence of CS. ROC curve analysis highlighted the superior predictive power of ASQoL (AUC = 0.97) and BASDAI (AUC = 0.92) for identifying CS in this patient population. Comparing these scores within the same sample population revealed that ASQoL, which directly reflects quality of life, is a more robust discriminator for CS than purely inflammatory scores like ASDAS-CRP. This finding emphasizes the need for integrating quality-of-life assessments into routine evaluations for a more holistic understanding of patient suffering.

CS is an important factor in non-inflammatory pain components in rheumatic diseases. Impaired peripheral and central pain processing is also exacerbated by inflammation. Neuroimaging studies have shown abnormalities in several functional brain networks and their interconnections in SpA [28]. Meanwhile, in the pathogenesis of CS in rheumatic diseases, the effect of vasoactive substances and immune cells and proinflammatory cytokines on the dorsal horn of the spinal cord via nociceptive neurons is important. Studies in animals have demonstrated that nociceptive and sensory neurons, especially TNF-α, IL-17, IL-6, and IL-1β, receptors, cause an increase in C-fiber action potentials, causing CS and pain [29]. In patients with elevated BASDAI scores, it is important to evaluate for potential CS before designating the patient as a ’non-responder’ to their current treatment. The presence of CS can result in inflated BASDAI scores, which may consequently affect the treatment strategy for axSpA [30]. In addition, if CS is detected in patients who may be considered non-responsive, non-pharmacological strategies such as exercise and electrotherapy as well as pharmacological treatments, such as pregabalin and duloxetine, should be utilized when deemed necessary [31].

There are several limitations of our study. While a sample size of 120 patients is acceptable, a larger cohort may increase the power of the study and the generalizability of the findings. Prospective randomized controlled studies to be conducted in this field are thought to make an important contribution due to the cross-sectional nature of our study.

CS is common in patients with SpA receiving biological therapy, with significant associations observed between CS, higher disease activity, and poorer quality of life. The findings underscore the need for routine CS assessments to better understand and manage persistent symptoms in SpA. Beyond SpA, CS has been implicated in other chronic diseases such as fibromyalgia, rheumatoid arthritis, and osteoarthritis, where it contributes to heightened pain perception and reduced quality of life. Incorporating insights from these conditions could further refine the management strategies for SpA. To identify patients with CS in routine practice, clinicians can focus on basic history and symptom patterns, such as disproportionate pain intensity, fatigue, and sleep disturbances, and also perform a musculoskeletal examination to assess tenderness in non-inflammatory areas, providing practical effectiveness in differentiating patients with CS.

While this study highlights the potential of CS-focused evaluations to improve treatment outcomes, it is essential to recognize that CS is not the sole determinant of patient outcomes. A holistic approach addressing inflammation, structural damage, and lifestyle factors, alongside tailored interventions for CS, is crucial. Future research, including validation assays, is needed to confirm whether routine CS assessments and subsequent targeted treatments consistently enhance management strategies in SpA and other rheumatic diseases.

## 5. Conclusions

Central sensitization (CS) is a significant factor contributing to persistent pain and reduced quality of life in patients with spondyloarthritis (SpA) receiving biological therapy. This study underscores the necessity of incorporating CS assessments into routine clinical evaluations to identify patients who may benefit from tailored interventions targeting non-inflammatory pain mechanisms. By identifying ASQoL and BASDAI scores as independent predictors of CS, our findings highlight the importance of addressing quality of life and disease activity in SpA management. Future research should focus on developing and validating integrative approaches that combine pharmacological and non-pharmacological strategies to optimize the outcomes for patients affected by CS.

## Figures and Tables

**Table 1:** Socio-demographic characteristics, clinical features, and comorbidity of the patients with axial spondyloarthritis based on the Central Sensitization Inventory (CSI) score.

	All Patients (n = 120)	CSI < 40 (n = 71)	CSI ≥ 40 (n = 49)	*p* Value	
Age (years) (mean ± SD)	43.9 SD 9.24	45.02 SD 8.53	42.26 SD 10.05	0.108	t
Female, gender, n (%)	49 (%40.8)	25 (%35.2)	24 (%49.0)	0.132	X^2^
Male, gender, n (%)	71 (%59.2)	46 (%74.8)	25 (%51.0)	0.132	X^2^
Marital status, married, n (%)	103 (%85.8)	62 (%87.3)	41 (%83.7)	0.573	X^2^
Education of high school and above, n (%)	62 (%52.5)	39 (%54.9)	24 (%49)	0.521	X^2^
Current smoker, n (%)	17 (%14.2)	10 (%14.1)	7 (%14.3)	0.590	X^2^
Hypertension, n (%)	4 (%3.3)	4 (%5.6)	0	0.144	*
Diabetes mellitus, n (%)	2 (%1.7)	2 (%2.8)	0	0.513	*
Weight (kg) (mean ± SD)	77.2 SD 11.4	78.9 SD 11.3	74.8 SD 11.3	0.057	t
Height (cm)	170 (165–178)	170 (165–178)	170 (165–175)	0.363	m
BMI (kg/m2)	26,6 (24–29.1)	25.8 (23.9–29.3)	26.7 (24.1–28.6)	1.000	m
Obesity (BMI ≥ 30 kg/m^2^), n (%)	32 (%26.7)	18 (%25.4)	14 (%28.6)	0.695	X^2^
Radiographic axSpA, n (%)	87 (%72.5)	51 (%71.8)	36 (%73.5)	0.843	X^2^
Non-radiographic axSpA, n (%)	33 (%27.5)	20 (%28.2)	13 (%26.5)	0.843	X^2^
Morning stiffness duration (minute)	36 (12–60)	24 (12–36)	72 (36–84)	<0.001	m
Hip involvement n (%)	29 (%24,2)	15 (%21.1)	14 (%28.6)	0.349	X^2^
History of uveitis n (%)	15 (%12.5)	9 (%12.7)	6 (%12.2)	0.944	X^2^
History of IBD, n (%)	8 (%6.7)	2 (%2.8)	6 (%12.2)	0.062	*
History of psoriasis, n (%)	14 (%11.7)	7 (%9.9)	7 (%14.3)	0.458	X^2^
Family history of SpA, n (%)	29 (%24.2)	18 (%25.4)	11 (%22.4)	0.714	X^2^
Symptoms duration (month)	162 (114–219)	170 (135–230)	140 (98–205)	0.024	m
Disease duration (month)	129 (92–172)	134 (105–167)	110 (80–176)	0.046	m
Time delay in diagnosis (month)	20 (12.5–38)	25 (13–48)	15 (12–25)	0.035	m
CRP (mg/dL)	4.1 (3.0–10)	4.18 (3.0–9.17)	3.90 (3.02–10.0)	0.571	m
ESR (mm/h)	20 (6–36)	20 (6–34)	21 (6–37)	0.687	m
Use of biological drugs duration (month)	74 (54–100)	75 (54–100)	66 (58–98)	0.781	m
*Biologic drugs used*					
Adalimumab, n (%)	34 (%28.3)	17 (%23.9)	17 (%34.7)	0.201	X^2^
Golimumab, n (%)	32 (%26.7)	22 (%31)	10 (%20.4)	0.198	X^2^
Infliximab, n (%)	5 (%4.2)	1 (%1.4)	4 (%8.2)	0.069	*
Etanercept, n (%)	16 (%13.3)	13 (%18.3)	3 (%6.1)	0.054	X^2^
Sertolizumab, n (%)	10 (%8.3)	6 (%8.5)	4 (%8.2)	0.955	*
Sekukinumab, n (%)	23 (%19.2)	12 (%16.9)	11 (%22.4)	0.448	X^2^
Patients using ≥ 3 biological drugs, n (%)	13 (%10.8)	1 (%1.4)	12 (%24.5)	<0.001	X^2^

t: Independent sample *t* test; m: Mann–Whitney u test; X^2^: Chi-square test; * Fisher’s exact test. Values are presented as the mean ± standard deviation, number (%), or median (interquartile range). Abbreviations: CSI, Central Sensitization Inventory; SD, standard deviation; BMI, body mass index; axSpA, axial spondyloarthritis; IBD, inflammatory bowel disease; SpA, spondyloarthritis; CRP, C-reactive protein; ESR, erythrocyte sedimentation rate.

**Table 2:** Disease-related clinical variables and laboratory findings of the patients with axial spondyloarthritis based on the Central Sensitization Inventory (CSI) score.

	All Patients (n = 120)	CSI < 40 (n = 71)	CSI ≥ 40 (n = 49)	*p* Value	
*Disease-related clinical variables*					
CSI Part A (mean ± SD)	35.44 SD 16.25	23.83 SD 7.87	52.27 SD 8.71	<0.001	t
BASDAI score	3.4 (2.5–5.4)	2.6 (1.8–3.4)	5.5 (5.0–6.6)	<0.001	m
ASDAS-CRP (mean ± SD)	2.80 SD 0.79	2.50 SD 0.71	3.24 SD 0.70	<0.001	t
ASDAS-ESR (mean ± SD)	2.85 SD 0.85	2.55 SD 0.82	3.30 SD 0.69	<0.001	t
BASFI score	3.5 (2.1–5.8)	2.4 (1.6–3.8)	5.7 (4.0–6.4)	<0.001	m
BASMI score	3.05 (2.0–4.9)	3.0 (2.0–4.5)	3.4 (2.0–5.0)	0.957	m
MASES score	4.0 (2.0–7.0)	3.0 (1.0–6.0)	5.0 (3.0–7.0)	0.005	m
ASQoL score	6 (4–11)	5 (3–6)	12 (10–13)	<0.001	m
VAS-fatigue score	5 (3–7)	40 (30–50)	70 (60–80)	<0.001	m
VAS-pain score	40 (30–60)	30 (20–40)	60 (50–80)	<0.001	m
PtGA score	55 (40–70)	50 (30–70)	60 (50–70)	0.005	m
PGA score	50 (30–60)	50 (30–60)	50 (50–60)	0.017	m
*Laboratory findings*					
25-hydroxy-vitamin D [25(OH)D] (ng/mL)	20 (15.9–25.4)	20.0 (16.5–26.8)	19.9 (15.0–25.0)	0.248	m

t: Independent sample *t* test; m: Mann–Whitney u test. Values are presented as the mean ± standard deviation or median (interquartile range). Abbreviations: CSI, Central Sensitization Inventory; SD, standard deviation; BASDAI, Bath Ankylosing Spondylitis Disease Activity Index; ASDAS, Ankylosing Spondylitis Disease Activity Score; CRP, C-reactive protein; ESR, erythrocyte sedimentation rate; BASFI, Bath Ankylosing Spondylitis Functional Index; BASMI, Bath Ankylosing Spondylitis Metrology Index; MASES, Maastricht Ankylosing Spondylitis Enthesitis Score; ASQoL, Ankylosing Spondylitis Quality of Life; VAS, Visual Analog Scale; PtGA, Patient Global Assessment; PGA, Physician Global Assessment.

**Table 3:** Correlation of disease-related clinical variables with Central Sensitization Inventory (CSI) score.

	CSI-A r
Age, years	−0.172
Disease duration, month	−0.164
Symptoms duration, month	−0.186 *
BASDAI score	0.774 **
ASDAS-CRP	0.538 **
ASDAS-ESR	0.479 **
ASDAS-CRP	0.538 **
BASFI score	0.509 **
BASMI score	−0.047
MASES score	0.305 **
ASQoL score	0.839 **
VAS-fatigue score	0.666 **
VAS-pain score	0.630 **
PtGA score	0.374 **
PGA score	0.337 **
ESR, mm/h	0.054
CRP, mg/dL	0.025

Abbreviations: CSI, Central Sensitization Inventory; BASDAI, Bath Ankylosing Spondylitis Disease Activity Index; ASDAS, Ankylosing Spondylitis Disease Activity Score; CRP, C-reactive protein; ESR, erythrocyte sedimentation rate; BASFI, Bath Ankylosing Spondylitis Functional Index; BASMI, Bath Ankylosing Spondylitis Metrology Index; MASES, Maastricht Ankylosing Spondylitis Enthesitis Score; ASQoL, Ankylosing Spondylitis Quality of Life; VAS, Visual Analog Scale; PtGA, Patient Global Assessment; PGA, Physician Global Assessment. * *p* < 0.05. ** *p* < 0.01.

**Table 4:** The independent effects of some possible predictors in relation to CSI (CSI ≥ 40) according to univariate/multivariate analysis.

	Univariate		Multivariate	
	OR (95% CI)	*p* Value	OR (95% CI)	*p* Value
Patients using ≥3 biological drugs	22.703 (2.840–181.456)	0.003	85.173 (0.657–11,038.403)	0.073
BASDAI score	3.943 (2.504–6.210)	<0.001	2.221 (1.134–4.350)	0.020
ASDAS-CRP	4.323 (2.241–8.340)	<0.001		
ASDAS-ESR	3.529 (1.948–6.394)	<0.001		
BASFI score	1.792 (1.428–2.247)	<0.001		
MASES score	1.164 (1.029–1.316)	0.016		
ASQoL score	3.165 (2.034–4.924)	<0.001	3.030 (1.755–5.230)	<0.001
VAS-fatigue score	1.072 (1.046–1.100)	<0.001		
VAS-pain score	1.077 (1.050–1.106)	<0.001		
PtGA score	1.028 (1.009–1.048)	0.004		
PGA score	1.023 (1.004–1.043)	0.020		

Abbreviations: CSI, central sensitization inventory; OR, odds ratio; CI, confidence interval; BASDAI, Bath Ankylosing Spondylitis Disease Activity Index; ASDAS, Ankylosing Spondylitis Disease Activity Score; CRP, C-reactive protein; ESR, erythrocyte sedimentation rate; BASFI, Bath Ankylosing Spondylitis Functional Index; MASES, Maastricht Ankylosing Spondylitis Enthesitis Score; ASQoL, ankylosing spondylitis quality of life; VAS, Visual Analogue Scale; PtGA, Patient Global Assessment; PGA, Physician Global Assessment.

## Data Availability

Data supporting the findings of this study are available from the corresponding author upon reasonable request. Data sharing complies with institutional and ethical guidelines.
